# Supplementary material for: Opposite effects of systemic and local conditional CD11c+ myeloid cell depletion during bleomycin‐induced inflammation and fibrosis in mice
Source: Immun Inflamm Dis. 2024 Nov 24;12(11):e70042. doi: 10.1002/iid3.70042 (PMC11586507; doi:10.1002/iid3.70042)
Supplement: Supplementary file 2 — Supporting information. [file IID3-12-e70042-s001.pdf]

Opposite effects of systemic and local conditional CD11c+ myeloid cell depletion during bleomycin-induced inflammation and fibrosis in mice

Gabriel Augusto Oliveira Lopes[1], Braulio Henrique Freire Lima[1,2], Camila Simões Freitas[1], Andiara Cardoso Peixoto[1], Frederico Marianetti Soriani[3], Geovanni Dantas Cassali[4], Bernhard Ryffel[5], Mauro Martins Teixeira[2], Fabiana Simão Machado[6], Remo Castro Russo[1#]

[1]*Laboratory of Pulmonary Immunology and Mechanics, Department of Physiology and Biophysics, Institute of Biological Sciences, Universidade Federal de Minas Gerais, Belo Horizonte, Minas Gerais, Brazil;*

[2]*Department of Biochemistry and Immunology, Institute of Biological Sciences, Universidade Federal de Minas Gerais, Belo Horizonte, Minas Gerais, Brazil;*

[3]*Department of Genetics, Ecology, and Evolution, Institute of Biological Sciences, Universidade Federal de Minas Gerais, Belo Horizonte, Minas Gerais, Brazil;*

[4]*Department of General Pathology, Institute of Biological Sciences, Universidade Federal de Minas Gerais, Belo Horizonte, Minas Gerais, Brazil;*

[5]*Experimental and Molecular Immunology and Neurogenetics, University of Orleans, CNRS UMR7355, Orleans, France;*

[6]*Laboratory of Immunoregulation of Infectious Diseases, Department of Biochemistry and Immunology, Institute of Biological Sciences, Universidade Federal de Minas Gerais, Belo Horizonte, Minas Gerais, Brazil.*

Running title: CD11c+ myeloid cells contribute to lung fibrosis.

#Author for correspondence: Prof. Remo C. Russo, Laboratory of Pulmonary Immunology and Mechanics, Department of Physiology and Biophysics, Institute of Biological Sciences, Universidade Federal de Minas Gerais (UFMG), Avenida Antônio Carlos, 6627, Pampulha, 31270-901, Belo Horizonte, MG, Brazil (e-mail: remo@ufmg.br). Orcid number: 0000-0002-1715-3834.

Data Availability Statement: The original contributions presented in the study are included in the article/supplementary material. Further inquiries can be directed to the corresponding author.

Abstract:

Rationale: Elevated levels of CD11c+ myeloid cells are observed in various pulmonary disorders, including Idiopathic Pulmonary Fibrosis (IPF). Dendritic cells (DCs) and macrophages (MΦ) are critical antigen-presenting cells (APCs) that direct adaptive immunity. However, the role of CD11c+ myeloid cells in lung extracellular matrix (ECM) accumulation and pulmonary fibrosis is poorly understood.

Objective: We aimed to investigate the impact of depleting CD11c+ myeloid cells, including DCs and macrophages, during bleomycin-induced pulmonary fibrosis in mice.

Methods: We used a diphtheria toxin (DTx) receptor (DTR) transgenic mouse model (CD11c-DTR-Tg) to deplete CD11c+ myeloid cells through two methods: Systemic Depletion (SD) via intraperitoneal injection (i.p.) and local depletion (LD) via intranasal instillation (i.n.). We then assessed the effects of CD11c+ cell depletion during bleomycin-induced lung inflammation and fibrosis.

Results: Fourteen days after bleomycin instillation, there was a progressive accumulation of myeloid cells, specifically F4/80-MHCII+CD11c+ DCs and F4/80+MHCII+CD11c+ MΦ, preceding mortality and pulmonary fibrosis. Systemic depletion of CD11c+ DCs and MΦ via i.p. DTx administration in CD11c-DTR-Tg mice protected against bleomycin-induced mortality and pulmonary fibrosis compared to wild-type (WT) mice. Systemic depletion reduced myeloid cells, airway inflammation (total leukocytes, neutrophils, and CD4+ lymphocytes in bronchoalveolar lavage (BAL), inflammatory and fibrogenic mediators, and fibrosis-related mRNAs (Collagen-1α1 and α-SMA). Increased anti-inflammatory cytokine IL-10 and CXCL9 levels were observed, resulting in lower lung hydroxyproline content and Ashcroft fibrosis score. Conversely, local depletion of CD11c+ cells increased mortality by acute leukocyte influx (predominantly neutrophils, DCs, and MΦ in BAL) correlated to IL-1β, with lung hyper-inflammation and early fibrosis development.

Conclusion: Systemic depletion of CD11c+ cells confers protection against inflammation and fibrosis induced by Bleomycin, underscoring the significance of myeloid cells expressing F4/80-MHCII+CD11c+ DCs and F4/80+MHCII+CD11c+ MΦ orchestrating the inflammatory milieu within the lungs, potentially as a source of cytokines sustaining pulmonary chronic inflammation leading to progressive fibrosis and mortality.
Keywords: Myeloid, Dendritic cell, Macrophage, CD11c, α-X Integrin, fibrosis.

Introduction:

Pulmonary fibrosis represents the common end-stage manifestation among a heterogeneous array of lung disorders, termed Interstitial Lung Diseases (ILDs). These ILDs are categorized together owing to shared clinical presentations, pathological classifications, physiological and radiographic changes, albeit with varying degrees of morbidity[1]. Among these disorders, Idiopathic Pulmonary Fibrosis (IPF) stands out as a lethal chronic fibro-proliferative disease of obscure etiology and lacking effective therapeutic interventions. It is typified by an aberrant accumulation of collagen within the pulmonary parenchyma. Clinical manifestations of IPF encompass deteriorating dyspnea, progressive decline in lung volume, and impaired gas exchange[2-4]. IPF exhibits a prevalence of 30 cases per 100,000 individuals, with an annual incidence of 34,000 new cases in the United States[5]. Typically, mortality in IPF stems from the progressive decline in lung function over time, culminating in respiratory failure. On average, individuals diagnosed with IPF have a survival expectancy of 3 to 5 years post-diagnosis[2]. IPF is a progressive and fatal interstitial disease with limited therapies. Previously, therapy for IPF predominantly was the chronic administration of corticosteroids, often supplemented with immunosuppressive agents. However, the emergence of adverse effects during the treatment course significantly constrains its efficacy[3, 6-8]. However, their efficacy in treating IPF is limited. Presently, the therapy for IPF is based on anti-fibrotics. Although Pirfenidone and Nintedanib are currently approved for treating idiopathic PF (IPF), patients still have high mortality rates and a median survival duration of only 3–5 years[9-12]. Regarding the origin of IPF, extensive research efforts have delved into elucidating the preceding events that trigger the abnormal scarring of lung tissue. Despite these efforts, the ontogenesis of IPF remains controversial and poorly understood[13, 14]. Presently, several hypotheses are proposed regarding the origin of IPF: (i) Fibrosis may arise due to a breakdown in communication or crosstalk between epithelial cells and fibroblasts, leading to collagen deposition independent of prior pulmonary inflammation. (ii) Alternatively, it can develop via an inflammatory pathway, wherein fibrosis results from preceding alveolitis and tissue damage[13-18].

The lung exhibits specific characteristics concerning the innate immune organization in health conditions and response upon stimulation. Myeloid cells are extensively distributed throughout the healthy lung and play pivotal roles in various respiratory disorders. They have been unequivocally implicated in the pathogenesis of chronic diseases[19, 20]. Myeloid cells such as Macrophages (MΦ) and Dendritic cells (DCs) serve as professional antigen-presenting cells (APCs), possessing a distinct capacity to initiate and regulate specific immune responses. The presence of a range of MΦ and DCs populations in the lungs constitutes an innate and remarkably responsive sentinel network strategically positioned in and around the airways[19-24]. Through the continuous conveyance of antigenic information from the airways to the pulmonary lymph nodes,

the APCs exert a profound influence on immune responses within the lungs, capable of both positive and negative modulation[19-22, 25]. APCs such as DCs and MΦ are primarily distinguished by their abundant expression of MHCII (Major Histocompatibility Complex class II – HLA-DR) and the integrin CD11c as surface markers predominantly expressed by myeloid cells. Additionally, MΦ express monocyte lineage markers (F4/80 and Ly6C), whereas DCs do not[19, 20]. The integrin CD11c (integrin α-X subunit, also referred to as leucocyte surface antigen p150, αXβ2, and CR4) is a transmembrane glycoprotein weighing 145-150 kDa, belonging to the β2 integrin family[26]. CD11c is predominantly expressed by cells of myeloid origin and serves as markers for defining leukocyte sub-populations, particularly since they are primarily expressed by macrophages and DCs[19, 20, 26]. As an integrin, CD11c plays crucial roles in regulating three critical aspects of immune cell function that contribute to and modulate immune responses: (i) orchestrating leukocyte recruitment to sites of inflammation, (ii) mediating the formation of cell-cell contacts, and (iii) triggering downstream effects on cellular signaling pathways of activation[26]. In lung sites, the populations of APCs that exhibit high co-expression of MHCII and CD11c primarily consist of Alveolar Macrophages (AMΦ) and Myeloid Dendritic Cells (mDCs), whereas Plasmacytoid Dendritic Cells (pDCs) do not typically display this co-expression pattern[19-22, 25].

Myeloid cells present in the airways and lung parenchyma possess the potential to sustain chronic inflammation through continual lymphocytic infiltration while also regulating cytokine and chemokine levels[20, 22, 27-30]. The expression of cytokines and chemokines during aberrant chronic inflammation results in the persistent accumulation of myeloid cells in the lungs. This sustained presence of myeloid cells leads to chronic activation of fibroblasts, producing abnormal tissue repair responses[27, 30-36]. In the context of ILDs, various subsets of Mϕ and mDCs have been documented in the bronchoalveolar lavage fluid (BALF) and lungs from IPF patients compared to healthy control[27, 37-40]. Despite clinical evidence indicating a correlation between elevated numbers of Macrophages (MΦ) and myeloid Dendritic Cells (mDCs) and the extent of pulmonary fibrosis in Idiopathic Pulmonary Fibrosis (IPF), the precise role of CD11c+ myeloid DCs and MΦ populations in the context of pulmonary fibrosis remains uncertain[41, 42], and was the objective of this investigation. Here, we investigated the involvement of myeloid CD11c+ Dendritic Cells (DCs) and Macrophages (MΦ) in advancing experimental pulmonary fibrosis induced by Bleomycin. Our findings revealed an augmentation in the myeloid CD11c+ populations, including MΦ (F4/80+ MHCII+ CD11c+) and DCs (F4/80- MHCII+ CD11c+), within the fibrotic lungs of bleomycin-treated mice, exhibiting a correlation with the progression of pulmonary fibrosis. To further elucidate the role of CD11c+ MΦ and DCs populations in experimental pulmonary fibrosis, conditional depletion of myeloid CD11c+ cells was achieved using a diphtheria toxin (DTx) receptor (DTR) transgenic mouse model (CD11c-DTR-Tg)[43]. Through two distinct protocols,

conditional CD11c+ systemic depletion and conditional CD11c+ local depletion via DTx administration, we investigated the impact on bleomycin-induced pulmonary fibrosis. Conditional systemic depletion of CD11c+ DCs and Macrophages (MΦ) using CD11c-DTR-Tg mice conferred protection against bleomycin-induced mortality and pulmonary fibrosis. This protection was associated with reduced influx of myeloid F4/80-MHCII+CD11c+ DCs and F4/80+MHCII+CD11c+ MΦ into the airways, accompanied by diminished levels of cytokines and chemokines, as well as attenuated airway inflammation and fibrogenesis. However, conditional local depletion of CD11c+ cells proved deleterious, exacerbating lung hyper-inflammation induced by Bleomycin, with early fibrosis development. These findings suggest that CD11c+ DCs and MΦ mediate chronic lung inflammation, sustaining tissue chemokine and cytokine levels that precede pulmonary fibrosis induced by Bleomycin in mice. Consequently, therapeutic strategies targeting CD11c+ DCs and MΦ may present a novel approach to treating lung fibroproliferative diseases such as IPF.

Material and methods:

Animals: C57Bl/6j (WT) and CD11c diphtheria toxin (DTx) receptor (DTR) transgenic mice CD11c-DTR-Tg (B6.FVB-Tg (Itgax-DTR/EGFP)57$^{Lan/J}$)[43, 44], were bred in animal facility from Laboratório de Imunofarmacologia/UFMG at Instituto de Ciências Biológicas/UFMG. Mice were grouped into three to five animals per cage in a ventilated rack and kept under controlled light conditions in the animal facility of the Laboratory of Pulmonary Immunology at Instituto de Ciências Biológicas/UFMG, under pathogen-free conditions with filtered water and food ad libitum.

Conditional depletion of CD11c$^+$ myeloid cells in vivo: Conditional depletion of CD11c+ myeloid cells in vivo was achieved using Diphtheria Toxin (DTx) (D-0564; Sigma-Aldrich, St. Louis, MO). DTx was reconstituted to a concentration of 2 mg ml−1 in sterile water and subsequently diluted to 2.5 µg ml−1 in sterile saline. CD11c-DTR-Tg mice were subjected to conditional depletion, following previously established protocols[43, 44], through two distinct methods: (i) Systemic depletion (SD) involved intraperitoneal injection (i.p.) of diphtheria toxin (DTx) at a dosage of 5 ng/g of body weight (Figure 2A); and (ii) Local depletion (LD) entailed intranasal instillation (i.n.) of diphtheria toxin (DTx) at a dosage of 1 ng/g of body weight (Figure 6A), diluted in sterile PBS, and both administered 24 hours prior to the challenge with bleomycin or control mice.

Bleomycin-induced lung injury and fibrosis in mice: Male wild-type (WT) and CD11c-DTR mice aged eight to ten weeks were utilized in a bleomycin-induced lung injury and fibrosis model, conducted in accordance with previously established procedures[45-49]. Mice mortality across different groups was monitored daily over 21 days. In brief, a single intranasal instillation of either a 40 µL injection containing 3.75 mg/kg of bleomycin sulfate (Blenoxane; Bristol-Meyers Squibb) per kg of body weight (equivalent to 3.75 Units of body weight) diluted in PBS, or PBS alone (vehicle), was administered. All experiments were conducted in compliance with institutional guidelines and approved by the Brazilian animal ethics committee CETEA/UFMG (Protocol number 232/2012), adhering to national and international laws.

Assessment of airway and alveolar leukocytes. Bronchoalveolar lavage (BAL) was conducted to retrieve leukocytes from the alveolar space, following established protocols, as described previously[45-49]. The trachea was exposed, and a polyethylene catheter (BD Biosciences) with a 1.7-mm outside diameter was inserted. Bronchoalveolar lavage (BAL) was performed by washing the lungs three times with two separate 1-ml aliquots of PBS. Then, BAL samples were centrifuged at 600 x g for 10 minutes at 4°C. The resulting cell pellet was resuspended in PBS, and the total number of leukocytes was determined by cell counting after dilution in Türk's solution (Merck

Millipore) using a Neubauer chamber (Blaubrand). Differential BAL cell counts were obtained from Cytospin preparations (Shandon III), and the percentage of leukocyte populations stained with May-Grünwald-Giemsa (Laborclin) was evaluated under an optical microscope. Additionally, leukocyte sub-populations were determined by FACS analysis.

FACS analysis of airway leukocytes: Leukocytes obtained from BAL were stained with fluorochrome-conjugated monoclonal antibodies, including anti-CD3, anti-CD4, anti-CD8, anti-γδTCR, anti-F4/80, anti-MHCII, anti-CD11c, and anti-GR1, or isotype controls (BD Pharmigen™). Subsequently, they were acquired using a FACSCanto II cytometer (BD Biosciences) using BD FACSDiva software (BD Biosciences), following established protocols as described previously[45-47]. Prior to staining with leukocyte antibodies, BAL cells underwent hypotonic lysis to remove residual erythrocytes. Subsequently, the cell suspensions were incubated with Fc Block antibodies (BD Pharmigen™). After staining, cells were fixed with 4% paraformaldehyde, and 100 thousand events were acquired in BAL samples. The gating strategy initially involved the Boolean gate of myeloid populations, singlets, and time. Analyses were performed using FlowJo V10.1 software (Tree Star, Ashland, OR, USA). The populations were determined in histograms of the constitutive marker. The leukocyte and phenotypic populations were calculated based on the expression of surface markers: Neutrophils (GR1+ F4/80-), T CD4 lymphocytes (CD3+CD4+), T CD8 lymphocytes (CD3+CD8+), γδ lymphocytes (CD3+γδTCR+), Macrophages (F4/80+ MHCII+ CD11c+), and Dendritic Cells (F4/80- MHCII+ CD11c+). The frequency, presented as a percentage of the analyzed population compared to the total acquired events, was utilized in the construction of graphs. The number of cells collected by BAL was adjusted based on the total cell counts.

Lung histopathology: The left lung was removed and fixed in 4% neutral phosphate-buffered formalin (pH 7.4), as we previously described[45-49]. The tissues underwent gradual dehydration in ethanol, followed by embedding in paraffin and cutting into 4 µm sections. These sections were then stained with H&E or Gomori's trichrome (Leica Biosystems) and examined under light microscopy by a pathologist in a blind manner. Semiquantitative histopathological analyses of inflammation were conducted as previously described[47, 49]. Briefly, ten random images per lung were acquired at 20x magnification, and the pulmonary inflammation score was recorded on a six-grade scale. A score of "0" corresponded to less than 1% of lung tissue area inflamed, while higher scores indicated increasing levels of inflammation. The hemorrhage score was based on a four-degree scale, ranging from the absence of hemorrhage to the presence of intense hemorrhage. For semiquantitative analysis of Gomori's trichrome-stained slices, the Ashcroft fibrosis score was determined according to custom-designed criteria for evaluating lung fibrosis, as previously

described[46, 49]. Images of lung sections were captured using a digital camera (Optronics DEI-470) connected to a microscope (Olympus IX70) with magnifications of 40x or 100x. Scale bars of 100 μm were utilized for Gomori's trichrome images, while those of 200 μm were used for H&E images.

Lung hydroxyproline (OH-proline) quantification: Lung fragments were harvested for hydroxyproline quantification to assess collagen content in the lung tissue, following established protocols as previously described[46-49]. Fragments weighing 100 mg of lung tissue were homogenized in 0.2% saline, followed by freezing and lyophilization. The assay was conducted using the lyophilized material, which underwent alkaline hydrolysis in 300 μL of H_2O supplemented with 75 μL of 10M NaOH at 120°C for 20 minutes. Subsequently, an aliquot of 50 μL of the hydrolyzed tissue was combined with 450 μL of Chloramine T (Sigma-Aldrich) oxidizing reagent (comprising 0.056 M Chloramine T and 10% n-propanol (Rauter Química Ltda) in acetate-citrate buffer [pH 6.5]) and allowed to react for 20 minutes. A hydroxyproline standard curve was prepared using a similar approach. Color development was initiated by adding 500 μL of 1M p-dimethylaminebenzaldehyde (Sigma-Aldrich) diluted in n-propanol–perchloric acid (2:1 [vol/vol]). The absorbance was measured at 550 nm using a spectrophotometer (Emax; Molecular Devices).

Cytokine measurement in lung tissue. One hundred mg of lung tissue were homogenized in 1 mL of PBS containing protease inhibitors (Complete tablets, Roche Diagnostic; PMSF, Sigma-Aldrich) and 0.05% Tween 20 (Sigma-Aldrich), following previously established protocols[45-49]. Samples of lung homogenate or bronchoalveolar lavage fluid (BALF) were then centrifuged for 10 minutes at 3000 x g, and the supernatant was immediately used for assay at a 1:2 dilution in PBS. The chemokines CCL2/MCP-1, CCL3/MIP-1α, CCL4/MIP-1β, CCL5/RANTES, CCL11/Eotaxin, CXCL1/KC, and CXCL9/MIG, as well as the cytokines IL-1β, IL-6, IL-10, and active TGF-β1 levels, were quantified in lung homogenate and BALF samples using DuoSet ELISA kits (R&D Systems), following the manufacturer's instructions, as previously described[45-49].

RNA isolation and Quantitative Real-Time PCR: Lungs were harvested for analysis of fibrogenesis markers, precisely the expression of Collagen1α1 (Col1a1) and α-Smooth Muscle Actin (Acta2) mRNAs, using quantitative Real-Time PCR, following established protocols as previously described[45, 47, 49]. Total lung RNA was extracted using TRIzol (Invitrogen) according to the manufacturer's instructions. First-strand cDNA synthesis was performed using the High Capacity cDNA Reverse Transcription Kit (Applied Biosystems) with 2 μg of total RNA, following the manufacturer's instructions. Real-time quantitative PCR was carried out on an ABI PRISM

Step-One sequence-detection system (Applied Biosystems, Carlsbad, CA) using SYBR Green PCR Master Mix (Applied Biosystems). The amplification protocol consisted of one cycle at 95°C for 10 minutes, followed by 39 cycles at 95°C for 15 seconds and 60°C for 1 minute. The relative expression level of genes was determined using the $2^{(-deltaCt)}$ method, and the data were normalized by the expression levels of the 18S ribosomal subunit. All reactions were performed in duplicate. The following primers were used:

Collagen-1α1 (Col1a1):	*Forward:* 5'- ATG TTC AGC TTT GTG GAC CTC - 3'
	Reverse: 5'- GCA GCT GAC TTC AGG GAT GT - 3'
α-Smooth Muscle Actin (Acta2):	*Forward:* 5'- GAC ACC ACC CAC CCA GAG T - 3'
	Reverse: 5'- ACA TAG CTG GAG CAG CGT CT - 3'
18S:	*Forward:* 5'- CTC AAC ACG GGA AAC CTC AC - 3'
	Reverse: 5'- CGC TCC ACC AAC TAA GAA CG - 3'

Statistical Analysis: The graphs were plotted using Prism version 10.1.1 (GraphPad), and values are presented as mean ± standard error of the mean (SEM). One-way ANOVA followed by Tukey post-hoc test was used for parametric data. The non-parametric data were expressed as the mean ± standard error of the mean (SEM) values and analyzed by Kruskal-Wallis followed by Dunn's test. The Mantel-Cox log-rank test was used to determine differences between survival curves. Statistical significance was accepted when $P<0.05$. Statistical significance is indicated as *$p<0.05$, **$p<0.01$, and ***$p<0.001$.

Results:

Bleomycin-induced pulmonary inflammation and mortality in mice were associated with a progressive influx of CD11c+ myeloid dendritic cells (DCs) and macrophages (MΦ), which preceded the development of lung fibrosis.

Myeloid dendritic cells (DCs) and macrophages (MΦ) play pivotal roles in orchestrating inflammation, immunity, and tolerance, making them critical targets for immunotherapy. We further characterized the phenotype of myeloid cell influx into the airways in bleomycin-treated mice. To do this, myeloid DCs (F4/80- MHCII+ CD11c+) and MΦ (F4/80+ MHCII+ CD11c+) were kinetically analyzed by flow cytometry of lung single-cell suspensions prepared from bronchoalveolar lavage fluid after 3, 7, and 14 days following instillation. An increased number of total airway leukocytes was observed starting from day 3, peaking after 7 days, and remaining elevated until day 14 after bleomycin instillation compared to PBS-instilled control wild-type (WT) mice (Figure 1A). Bleomycin instillation progressively induced myeloid cell influx into the airways (Figure 1A), with DCs (F4/80- MHCII+ CD11c+) increasing from day 3 until day 14 and MΦ (F4/80+ MHCII+ CD11c+) increasing from day 7 until day 14 (Figure 1A), compared to PBS-instilled control mice. Therefore, myeloid DCs (F4/80- MHCII+ CD11c+) and MΦ (F4/80+ MHCII+ CD11c+) concomitantly increased after 7- and 14-days post-bleomycin instillation (Figure 1A). Bleomycin induced pronounced mice mortality after 7 days of instillation (Figure 1B), occurring in parallel with concomitant airway influx of DCs (F4/80- MHCII+ CD11c+) and MΦ (F4/80+ MHCII+ CD11c+) compared to PBS-instilled control mice (Figure 1A). After 21 days of bleomycin instillation, WT mice developed extended pulmonary fibrosis compared to PBS-instilled control mice, with increased hydroxyproline lung tissue content (Figure 1C), marked by a diffuse and dense pattern of fibrosis with airway collapse as visualized by Gomori's Trichrome staining (green) (Figure 1D), also exhibiting a high grade of pulmonary fibrosis as depicted by Ashcroft score (Figure 1E).

Collectively, Bleomycin induced pulmonary inflammation and mortality related to progressive and concomitant airway influx of CD11c+ myeloid DCs and MΦ starting 7 days after instillation, preceding chronic lung fibrosis in mice.

Conditional systemic depletion of CD11c+ myeloid cells attenuated lung inflammation due to reduced CD11c+ dendritic cells (DCs) and macrophages (MΦ) after 7 days of bleomycin instillation in CD11c-DTR-Tg mice.

Defining that CD11c+ myeloid DCs and MΦ airway influx are related to inflammation that precedes lung fibrosis, we sought to determine the role of CD11c+ myeloid cells in sterile innate

immune response, wherein chronic lung inflammation causes pulmonary fibrosis. Using CD11c-DTR-Tg mice, we systemically depleted CD11c+ myeloid cells via DTx intraperitoneal (i.p.) injection 24 hours before intranasal bleomycin instillation to evaluate the impact on leukocyte influx in airways after 7 days (Figure 2A), at the inflammatory peak. Bleomycin instillation induced increased airway leukocyte influx after 7 days in WT mice (Figure 2B), with elevated lymphocytes and neutrophil numbers in bronchoalveolar lavage (BAL), as shown by cytospin preparations (Figure 2C) compared to PBS-instilled mice. FACS analysis confirmed increased counts of CD3+CD4+ and CD3+γδ lymphocytes (Figure 2D), as well as GR1+F4/80- neutrophils (Figure 2E) in BAL of WT mice challenged with Bleomycin. Conditional systemic depletion using CD11c-DTR-Tg mice reduced total leukocyte numbers (Figure 2B), marked by a low influx of lymphocytes and neutrophils by cytospin (Figure 2C), primarily decreasing CD3+CD4+ lymphocytes (Figure 2D) and GR1+F4/80- neutrophils (Figure 2E) in BAL after 7 days of bleomycin instillation. Bleomycin induced CD11c+ myeloid cell influx into the airways, with myeloid DCs (F4/80- MHCII+ CD11c+) and MΦ (F4/80+ MHCII+ CD11c+) concomitantly increased after 7 days of instillation (Figure 2E), as previously shown (Figure 1A). Conditional systemic depletion of CD11c+ cells using CD11c-DTR-Tg mice abrogated the DCs (F4/80- MHCII+ CD11c+) and MΦ (F4/80+ MHCII+ CD11c+) BAL numbers induced by bleomycin instillation after 7 days (Figure 2E), confirming that CD11c+ depletion by DTx impacts the influx of DCs and MΦ populations in airways.

We further investigated the impact of bleomycin-induced tissue injury and inflammation in the context of conditional systemic depletion of CD11c+ myeloid cells. We evaluated lung histopathology using H&E stained slices, and inflammation was analyzed in all lung compartments (vascular, parenchymal, and airways) after 7 days of bleomycin instillation (Figure 3). Histopathological analysis revealed that Bleomycin induced lung injury 7 days after instillation in WT mice, with marked lung vascular inflammation, parenchymal leukocyte infiltration, and airway inflammation (Figure 3A), resulting in a multifocal pattern of lung inflammation (Figure 3B) and elevated overall score (Figure 3A) compared with PBS-instilled mice. However, conditional systemic depletion of CD11c+ cells using CD11c-DTR-Tg mice attenuated tissue injury and inflammation induced by Bleomycin compared to WT mice, with reduced inflammatory aspects of all lung compartments evaluated (Figure 3A) and increased preserved lung areas (Figure 3B).

Thus, this suggests that CD11c+ DCs and MΦ may contribute to the development of an inflammatory milieu in the lungs, with leukocyte influx and tissue injury induced by Bleomycin, and systemic depletion of CD11c+ cells was able to restrict these pathological manifestations in CD11c-DTR-Tg mice.

Conditional systemic depletion of CD11c+ myeloid cells changes the chemokine and cytokine profile, impacting the low expression of fibrogenic markers induced by Bleomycin after 7 days in CD11c-DTR-Tg mice.

Myeloid cells, such as dendritic cells (DCs) and macrophages (MΦ), are distributed throughout the airways and lung parenchyma. They can perpetuate chronic inflammation through sustained expression of cytokines and chemokines. This perpetuation contributes to aberrations in chronic inflammation, thereby fostering a persistent activation of fibroblasts. Consequently, this sustained activation results in an abnormal tissue repair process [27, 30-36]. Utilizing CD11c-DTR-Tg mice, we conducted a comprehensive investigation into the effects resulting from the conditional systemic depletion of CD11c+ myeloid cells on the chemokine and cytokine profile induced by bleomycin instillation over a 7-day period. Employing Enzyme-Linked Immunosorbent Assay (ELISA), we quantified the levels of CCL2, CCL3, CCL4, CCL5, CCL11, CXCL1, CXCL9, IL-1β, IL-6, IL-10, and TGF-β1 in bronchoalveolar lavage (BAL) and lung tissue samples. Bleomycin instillation in wild-type (WT) mice elicited significantly higher levels of CC and CXC chemokines in BAL fluid compared to phosphate-buffered saline (PBS)-instilled mice, as demonstrated by previous studies[30, 50], compared to PBS-instilled mice (Figure 4A). However, in the context of conditional systemic depletion of CD11c+ myeloid cells, there was a notable decrease in CC chemokines (CCL2, CCL3, CCL4, CCL5), along with comparable levels of CCL11, reduced levels of CXCL1, and elevated levels of CXCL9 in BAL fluid of CD11c-DTR-Tg mice compared to WT mice, 7 days post-bleomycin instillation (Figure 4A). Analyzing the inflammatory and fibrogenic cytokines in lung homogenates, we observed that bleomycin-induced increased levels of pro-inflammatory IL-1β and IL-6, alongside elevated levels of pro-resolutive IL-10 and pro-fibrogenic TGF-β1 at day 7 in WT mice compared to PBS-instilled mice (Figure 4B). However, the conditional systemic depletion of CD11c+ cells resulted in decreased levels of pro-inflammatory cytokine IL-6, reduced levels of pro-fibrogenic cytokines IL-1β and TGF-β1, but increased levels of pro-resolutive IL-10 in the lungs of bleomycin-challenged CD11c-DTR-Tg mice compared to WT mice (Figure 4B).

Given the observed differential expression of chemokines and cytokines in the lungs of CD11c-DTR-Tg mice compared to WT mice after bleomycin challenge (Figure 4A-B), particularly those associated with pro-fibrogenic actions (CCL2, CXCL1, IL-1β, and TGF-β1), we proceeded to investigate the expression of mRNA transcripts related to fibrogenesis (Figure 4C) via real-time PCR from lung tissue. We evaluated the expression of Collagen-1α1 (col1a1) and α-Smooth Muscle Actin (Acta2). Bleomycin-induced mRNA expression of Collagen-1α1 and α-Smooth Muscle Actin in WT mice after 7 days of instillation, indicative of fibroblast activity and extracellular matrix

(ECM) production, was abrogated by the conditional systemic depletion of CD11c+ myeloid cells using CD11c-DTR-Tg mice (Figure 4C).

Hence, it appears that CD11c+ dendritic cells (DCs) and macrophages (MΦ) may foster an inflammatory milieu in the lungs through chemokine and cytokine production induced by Bleomycin, sustaining leukocyte influx and fibroblast activity, phenomena attenuated by the systemic depletion of CD11c+ DCs and MΦ in CD11c-DTR-Tg mice.

The conditional systemic depletion of CD11c+ myeloid cells confers protection to CD11c-DTR-Tg mice against bleomycin-induced pulmonary fibrosis and mortality.

Bleomycin administration in wild-type (WT) mice elicited pronounced lung inflammation, along with concurrent expression of extracellular matrix (ECM) remodeling markers after a 7-day, and this inflammatory response was mitigated upon conditional systemic depletion of CD11c+ dendritic cells (DCs) and macrophages (MΦ) in CD11c-DTR-Tg mice. Therefore, we endeavored to ascertain the influence of CD11c+ myeloid cell depletion on initiating and preceding pulmonary fibrosis 21 days post-bleomycin instillation in mice. By analyzing lung tissue samples for hydroxyproline content, indicative of collagen deposition, we observed an increase in bleomycin-induced collagen accumulation 21 days post-instillation in WT compared to PBS-instilled mice, which was attenuated in CD11c-DTR-Tg mice upon early conditional systemic depletion of CD11c+ myeloid cells using diphtheria toxin (DTx) administered 24 hours prior to bleomycin challenge (Figure 5A). Furthermore, pulmonary fibrosis was quantified via assessment of the Ashcroft fibrosis score using Gomori's Trichrome stained lung sections. WT mice exhibited dense and diffuse interstitial lung fibrosis after 21 days of bleomycin instillation, characterized by elevated Ashcroft scores and marked alterations in pulmonary architecture, including alveolar septal collapse and large contiguous fibrotic masses, in contrast to PBS-instilled mice. However, these fibrotic changes were markedly reduced in CD11c-DTR-Tg mice subjected to early conditional systemic depletion of CD11c+ myeloid cells and challenged with Bleomycin, presenting with lower-grade Ashcroft scores and focal, smaller fibrotic masses interspersed with preserved lung parenchyma (Figure 5B-C). Progressive bleomycin-induced pulmonary fibrosis was associated with aberrant collagen accumulation within the lung parenchyma (Figure 5A-C). While bleomycin administration resulted in 90% mortality 21 days post-instillation, a 60% survival rate was achieved when CD11c+ myeloid cells were conditionally systemically depleted in CD11c-DTR-Tg mice (Figure 5D).

Taken together, these findings suggest that CD11c+ DCs and MΦ may contribute to chronic tissue remodeling, and early conditional systemic ablation of CD11c+ myeloid cells confers

protection against bleomycin-induced fibrotic disease, thereby enhancing survival in CD11c-DTR-Tg mice.

The conditional local depletion of CD11c+ myeloid cells exacerbates pulmonary hyperinflammation and increases mortality induced by Bleomycin in CD11c-DTR-Tg mice.

CD11c+ DCs and MΦ systemic depletion in CD11c-DTR-Tg mice protect mi e from inflammation and fibrosis, thereby enhancing survival. In light of these findings, we further explored the impact of conditional local depletion of CD11c+ myeloid cells on bleomycin-induced tissue injury and inflammation. To achieve this, we locally depleted CD11c+ myeloid cells using intranasal instillation of diphtheria toxin (DTx) in CD11c-DTR-Tg mice 24 hours prior to intranasal bleomycin instillation (Figure 6A), aiming to evaluate survival and leukocyte influx into the airways at the peak of inflammation after 7 days.

Conditional local depletion of CD11c+ myeloid cells using DTx in CD11c-DTR-Tg mice led to increased mortality, with Bleomycin inducing 100% mortality compared to a 60% survival rate observed in WT mice 8 days post-instillation (Figure 6B). Furthermore, conditional local depletion of CD11c+ myeloid cells resulted in increased total leukocyte numbers in bronchoalveolar lavage (BAL) after 7 days of bleomycin instillation (Figure 6C), primarily driven by an influx of GR1+F4/80- neutrophils (Figure 6E), but not lymphocytes (Figure 6D). Surprisingly, Bleomycin induced increased recruitment of CD11c+ myeloid DCs (F4/80- MHCII+ CD11c+) and MΦ (F4/80+ MHCII+ CD11c+) into the airways after 7 days of instillation in CD11c-DTR-Tg mice subjected to conditional local CD11c+ depletion, contrasting with WT mice (Figure 6E), suggesting that local CD11c+ depletion by DTx exacerbates leukocyte influx, particularly neutrophils, DCs, and MΦ populations in the airways.

Examining the chemokine and cytokine profile induced by Bleomycin after 7 days of instillation, we found that while WT mice exhibited significantly elevated levels of CC and CXC chemokines in BAL fluid, only CCL4 and CCL11 were increased in CD11c-DTR-Tg mice subjected to conditional local CD11c+ depletion (Figure 7A). Analysis of inflammatory and fibrogenic cytokines in lung homogenates revealed increased levels of IL-1β and low levels of IL-6 in CD11c-DTR-Tg mice, alongside similar levels of pro-resolutive IL-10 and pro-fibrogenic TGF-β1 compared to WT mice (Figure 7B). Additionally, bleomycin-induced mRNA expression of Collagen-1α1 and α-Smooth Muscle Actin, indicative of fibroblast activity and ECM production, was observed in WT mice similarly found in CD11c-DTR-Tg mice subjected to conditional local CD11c+ depletion (Figure 7C).

Histopathological analysis demonstrated marked lung injury induced by Bleomycin in WT mice after 7 days, characterized by lung vascular inflammation, parenchymal leukocyte infiltration, and airway inflammation, resulting in a multifocal pattern of lung inflammation and elevated overall score (Figure 8A and C). However, conditional local depletion of CD11c+ cells in CD11c-DTR-Tg mice exacerbated tissue injury and inflammation induced by Bleomycin compared to WT mice, with increased inflammatory scores of lung parenchyma and airways. diffuse lung inflammation and edema observed (Figure 8A and C). The pulmonary fibrosis was quantified at day 7 post-bleomycin instillation via assessment of the Ashcroft fibrosis score using Gomori's Trichrome stained lung sections (Figure 8B and D), as we previously described[48, 51]. WT mice exhibited lower-grade Ashcroft scores and focal fibrosis, with smaller airway thickness interspersed with preserved lung parenchyma at day 7. However, upon local CD11c depletion, there is a discrete increase in collagen accumulation and airway thickness with alveolar septal collapse, compared to WT, as depicted by the Ashcroft score (Figure 8B and D).

In summary, conditional local depletion of CD11c+ myeloid cells in CD11c-DTR-Tg mice exacerbates bleomycin-induced lung inflammation and tissue injury, contributing to the development of a hyperinflammatory lung milieu and early fibrosis, characterized by sustained production of chemokines and cytokines.

Discussion:

Idiopathic pulmonary fibrosis (IPF) stands as one of the most devastating progressive interstitial lung diseases. Its pathogenesis is characterized by an aberrant interplay among injured alveolar cells, leukocytes, and myofibroblasts, culminating in excessive fibroproliferation[52]. In various clinical studies, diverse clinical evidence has been documented delineating the extent of pulmonary fibrosis and the presence of distinct subsets of macrophages (MΦ) and myeloid dendritic cells (mDCs) in bronchoalveolar lavage fluid (BALF) and lung tissues from patients with interstitial lung diseases (ILDs), including IPF[27, 37-40]. Indeed, the precise role of CD11c+ DCs and MΦ in the context of pulmonary fibrosis remains elusive and subject to ongoing investigation[41, 42].

In our study, we delved into the role of myeloid CD11c+ DCs and MΦ in the progression of experimental pulmonary fibrosis induced by Bleomycin. Our findings revealed several key insights: (i) We observed that the pulmonary inflammation and mortality induced by bleomycin correlate with a progressive influx of CD11c+ myeloid DCs and MΦ into the airways, preceding the development of lung fibrosis; (ii) Conditional systemic depletion of CD11c+ myeloid cells resulted in the attenuation of lung inflammation, accompanied by a reduction in the numbers of CD11c+ DCs and MΦ following bleomycin instillation; (iii) This systemic depletion altered the chemokine and cytokine profile, leading to decreased expression of fibrogenic markers, thereby protecting against bleomycin-induced pulmonary fibrosis and mortality in CD11c-DTR-Tg mice; (iv) Conversely, conditional local depletion of CD11c+ myeloid cells exacerbated pulmonary hyper-inflammation, enhancing mortality in CD11c-DTR-Tg mice following bleomycin instillation. These findings collectively support the notion that myeloid cells expressing CD11c, including DCs (F4/80-MHCII+CD11c+) and MΦ (F4/80+MHCII+CD11c+), play critical roles in lung inflammation. These myeloid cells may serve as a source of cytokines and chemokines[30, 53, 54], thereby sustaining chronic inflammation that ultimately leads to progressive pulmonary fibrosis and mortality.

Myeloid cells such as MΦ and mDCs are naturally present in the airways and lung parenchyma during periods of health. However, they can initiate and perpetuate chronic inflammation through continuous lymphocytic infiltration and maintenance of cytokine and chemokine levels. This sustained inflammatory *milieu* contributes to the formation of *fibroblastic foci* and eventual tissue fibrosis[20, 22, 27, 28, 30]. Indeed, studies have consistently shown an abundance of MΦ and mDCs in BALF and lung tissues obtained from patients diagnosed with IPF[27, 37-40]. These findings underscore the significant role of myeloid cells in the pathogenesis of IPF and other ILDs. Our results indicate a progressive increase in the influx of CD11c+ myeloid cells, particularly DCs (F4/80-MHCII+CD11c+) and MΦ (F4/80+MHCII+CD11c+), following bleomycin instillation and preceding lung fibrosis in mice, are consistent with existing literature[37-39, 55, 56]. This parallels

the observed manifestation in humans with IPF, where there is an influx of macrophages and myeloid dendritic cells into the airways. These findings suggest a potential translational relevance of your experimental model to the human condition of IPF[27, 37-40]. Based on these results, we formulated a hypothesis to investigate whether conditional depletion of CD11c-expressing myeloid cells would influence the progression of inflammation preceding pulmonary fibrosis induced by Bleomycin in mice.

To gain deeper insights into the role of CD11c+ MΦ and mDCs populations during experimental pulmonary fibrosis, we opted to conditionally deplete myeloid CD11c+ cells using CD11c-DTR-Tg mice[43]. We aimed to evaluate the impact of conditional systemic (i.p.) and local (i.n.) depletion of CD11c-expressing myeloid cells on inflammation and fibrosis induced by Bleomycin in mice. Exploring the functions exhibited by various MΦ and DC subsets *in vivo* has encountered limitations due to difficulties depleting specific DC populations in murine models[43, 57]. However, in myeloid cell research emerges with the utilization of inducible cell ablation facilitated by transgenic expression of a high-affinity diphtheria toxin receptor (DTR)[43, 57]. However, some limitations of this model lead to caution in their interpretation, in which CD11c-DTR mice develop fatal fulminant myocarditis after local or systemic treatment with diphtheria toxin (DTx)[58]. Nevertheless, we did not detect any adverse reactions induced by systemic DTx administration in mice, such as heightened mortality. Instead, we observed protection against bleomycin-induced inflammation and fibrosis with CD11c systemic depletion, consistently with literature[53, 59, 60]. CD11c depletion using clodronate or CD11c-DTR local or systemically protects against lung fibrosis in preventive or therapeutic protocols of depletion[53, 59, 60]. Collectively, these results underscore the impact on the fibrogenesis process.

In the lungs, CD11c is predominantly expressed by myeloid cells such as alveolar macrophages (AMΦ) and myeloid dendritic cells (mDCs). These cells play crucial roles in maintaining tissue homeostasis, orchestrating immune responses, and contributing to the pathogenesis of various diseases[19-22, 25]. Several studies have aimed to elucidate the role of CD11c-expressing MΦ and mDCs in the context of pulmonary diseases. Mouse lung infection models have highlighted the importance of CD11c+ myeloid subsets in balancing immunity and inflammation. Depletion of CD11c+ MΦ or mDCs has been associated with compromised pathogen control efficiency and secondary tissue damage, underscoring their central role in immune response and pathology. In particular, the depletion of CD11c+ myeloid cells has been shown to exacerbate infection outcomes significantly. This is evidenced by increased bacterial loads and more severe pathology, ultimately leading to accelerated mortality during *Staphylococcus aureus* bacteremia[61]. Indeed, depletion of CD11c+ myeloid cells exacerbates acute lung injury (ALI), which is characterized by multi-organ damage. This suggests that CD11c+ myeloid cells play a crucial role

in mitigating the severity of ALI and in maintaining tissue homeostasis during acute inflammatory responses[62]. The presence of CD11c+ cells is essential for an effective immune response against influenza virus infection. Depletion of CD11c+ cells exacerbates aspects of infection, hindering the development of virus-specific CD8+ T cells. This impairment correlates with heightened clinical severity and delayed viral clearance[63]. In contrast to infectious models, in chronic non-infectious models such as asthma, the depletion of CD11c+ cells abolishes disease features in mice during chronic allergic lung disease. This suggests a distinct role for CD11c+ cells in the pathogenesis of chronic airway allergy compared to infectious models[64]. Similarly, in the context of sterile chronic airway inflammation, our findings demonstrated that conditional systemic depletion of CD11c+ myeloid cells attenuated lung injury and inflammation but not conditional local depletion. This attenuation was attributed to the impaired influx of CD11c+ DCs and MΦ after bleomycin instillation in CD11c-DTR-Tg mice. Consequently, there was a reduction in the degree of fibrosis and increased survival rates.

The persistent accumulation of myeloid cells in the lungs contributes to the activation of fibroblasts and abnormal tissue repair, a process sustained by the continued cytokine production[27, 30-36, 53, 54]. Tissue-related factors play a pivotal role in shaping the fate and function of immune cells, and the plasticity of leukocytes enables them to adapt to the tissue environment. This phenomenon, known as leukocyte tissue adaptation, involves the trafficking and residence of immune cells in specific tissue microenvironments[65], such as gut and lung mucosa. Indeed, myeloid cells in the lungs play a crucial role in discerning between natural stimulation from harmless microbiota or infections and exposure to noxious agents. They influence both leukocyte and tissue cells by serving as a continuous source of cytokines and chemokines. Additionally, myeloid cells promote the recruitment of specific leukocyte sub-populations, thereby maintaining chronic lymphocytic infiltration in the lung tissue[20, 22, 27, 28, 30]. In our hands, using the local depletion of CD11c+ myeloid cells previous to Bleomycin induced hyper-inflammation with signs of accelerated fibrogenesis, contrasting our systemic depletion finds *in vivo*. In contrast, Gibbons *et al.* (2011) demonstrated that local CD11c+ depletion using DTx or clodronate administered therapeutically in the fibrogenesis phase ameliorates bleomycin-induced lung fibrosis in mice[59]. However, we aimed to deplete CD11c+ cells with just one hit in the initial phase preceding the Bleomycin and evaluate the impact in the course of disease development. CD11c+CD103+ DCs might have an anti-inflammatory as well as anti-fibrotic nature in pulmonary fibrosis development[56], and CD11c+ local depletion may accelerate the disease progression. Thus, the local CD11C+ depletion in the early phase may lead to lung leukocyte maladaptation, favoring the recruitment of circulating myeloid cells and neutrophils, exacerbating the inflammatory response and greater lung injury, and consequently, mortality induced by bleomycin challenge.

Our findings demonstrate that conditional systemic depletion of CD11c+ myeloid cells effectively mitigated lung injury and inflammation following bleomycin instillation in CD11c-DTR-Tg mice. This intervention led to reductions in both leukocyte infiltration and cytokine levels. Specifically, the systemic depletion of CD11c+ myeloid cells resulted in decreased levels of CC chemokines (CCL2, CCL3, CCL4, CCL5) and CXC chemokine (CXCL1). However, local depletion of CD11c+ myeloid cells increases CCL4 and CCL11 in the lungs, which are critical for leukocyte chemoattraction and lung fibrosis[30, 50, 66]. CCL4 can interact with multiple receptors (CCR1, CCR3, and CCR5) meanwhile, CCL11 binds to CCR3, and those receptors are distributed in a range of leukocytes, including myeloid cells (DCs and MΦ) and granulocytes (neutrophils an eosinophils)[66]. Thus, supporting an inflammatory milieu with neutrophils, DCs, and MΦ recruitment induced by local CD11c depletion. Lung-resident macrophages, which include alveolar macrophages and interstitial macrophages (IMs), exhibit a high degree of diversity but coordinated chemokine signatures, highlighting the specialized roles of lung-resident macrophages, defined by their coordinated chemokine production and regulating immune cell influx[29], and contribute to lung fibrosis[30]. Concurrently, we observed lower counts of T CD4+ lymphocytes and neutrophils in bronchoalveolar lavage fluid (BALF) from bleomycin-challenged mice following conditional systemic depletion of CD11c+ myeloid cells but increased neutrophils in local depletion of CD11c+ myeloid cells. Myeloid cells serve as a significant source of chemokines[27, 30, 66], regulating the influx of leukocytes into the lungs. This process, if dysregulated, abnormal chemokine expression can lead to increased tissue damage and contribute to the development of fibrosis[30]. Indeed, myeloid cells not only regulate leukocyte influx but also act as producers of cytokines[29]. These cytokines play critical roles in activating both leukocytes and resident cells, thereby shaping tissue adaptation in response to tissue injury.

In the context of pulmonary fibrosis, myeloid cells, such as DCs and AMΦ, play a crucial role in producing profibrogenic cytokines, including IL-1β, IL-6, and TGF-β1, while also capable of producing the anti-inflammatory cytokine IL-10[35, 40, 42, 67, 68]. IL-1β[69, 70], IL-6[71], and TGF-β1[72] are potent inducers of fibrosis as they not only affect leukocytes but also activate resident cells such as fibroblasts and myofibroblasts, leading to proliferation, differentiation, and collagen production [73]. Our study demonstrated that conditional systemic depletion of CD11c+ myeloid cells effectively impacted cytokine levels following bleomycin instillation in CD11c-DTR-Tg mice. Specifically, there was a decrease in IL-1β, IL-6, and TGF-β1 levels in the lungs. However, local depletion of CD11c+ myeloid cells enhanced IL-1β levels correlated with increased GR1+ influx, early fibrosis, and mortality. Neutrophils are fundamental in the development of pulmonary fibrosis, contributing to the exacerbation and duration of inflammation, and fibrotic lung disease alters neutrophil trafficking and promotes neutrophil elastase and NETs release[48, 74, 75]. IL-1β is produced by

neutrophils and AMΦ[76, 77], and IL-1β-producing leukocyte traffic to the lungs in response to Bleomycin supports the concept that proinflammatory cytokine production by lung neutrophils and MΦ may contribute to the development of lung inflammation and fibrosis. In fact, bleomycin-induced chronic lung inflammation requires the inflammasome pathway and IL-1R1/MyD88 signaling, and IL-1β represents a critical effector of pathology and therapeutic target of inflammation and fibrosis[69]. Interestingly, neither systemic nor local depletion of CD11c+ myeloid cells altered the levels of the pro-resolving and anti-inflammatory cytokine IL-10[68, 78], following bleomycin instillation in CD11c-DTR-Tg mice. In addition to the observed decrease in profibrogenic cytokine levels, systemic depletion of CD11c+ myeloid cells was associated with reduced expression of fibrogenesis markers in the lungs[79]. Specifically, mRNA transcriptions of Collagen1α1 and α-Smooth Muscle Actin (α-SMA) were downregulated in systemic depletion of CD11c+ myeloid cells, indicating decreased fibroblast activity[79], but not observed by local depletion of CD11c+ myeloid cells. This suggests that the systemic depletion of CD11c+ myeloid cells may mitigate fibrogenesis by suppressing fibroblast activation and subsequent collagen production in the lung tissue. Taken together, these findings support that conditional systemic depletion of CD11c+ myeloid cells effectively reduced the levels of profibrogenic cytokines, potentially contributing to the attenuation of fibrogenesis progression and mortality.

Myeloid cells such as MΦ and mDCs are naturally present in the airways and lung parenchyma during homeostasis. However, under certain conditions, they have the potential to initiate and perpetuate chronic inflammation, contributing to the formation of fibroblastic foci and tissue fibrosis[20, 22, 27, 28]. Tissue repair is an essential protective response following injury, but it becomes problematic when injuries are repetitive or prolonged, potentially leading to tissue fibrosis. In this context, the myofibroblast-macrophage cell circuit plays a crucial role in either tissue healing or fibrosis. In "hot fibrosis," there is an excessive accumulation of extracellular matrix and scar formation, characterized by a significant presence of macrophages supporting fibroblast activity[80]. Our findings demonstrate that conditional systemic depletion of CD11c+ myeloid cells effectively mitigated the pathological state of excessive scarring following bleomycin instillation in CD11c-DTR-Tg mice, primarily due to impaired activity of CD11c+ DCs and MΦ. Moreover, this depletion disrupted the myofibroblast-macrophage cell circuit, potentially switching off the chronic activation of myofibroblasts by myeloid cells. This intervention may lead to a transition from "hot fibrosis," characterized by macrophage-supported fibroblast activity, to "cold fibrosis," which lacks macrophages and exhibits impaired wound healing[80].

Despite clinical evidence demonstrating a correlation between increased numbers of MΦ and mDCs and the degree of pulmonary fibrosis in IPF[27, 37-40], the specific role of CD11c+ myeloid populations in the context of pulmonary fibrosis remains unclear[41, 42], and was the objective of this

investigation. Here, we showed that systemic depletion of CD11c+ cells, not local depletion, confers protection against inflammation and fibrosis induced by Bleomycin, underscoring the significant role of myeloid cells expressing F4/80-MHCII+CD11c+ DCs and F4/80+MHCII+CD11c+ MΦ contributing with the initial phase of disease and progression. The single-dose model of Bleomycin has been instrumental in understanding fibrotic lung remodeling but fails to recapitulate several features of IPF and is self-resolutive after 2 months[81, 82]. However, repetitive intratracheal bleomycin results in marked lung fibrosis with prominent alveolar epithelial cell hyperplasia, resembling the IPF features [81]. Thus, future experiments on the depletion of CD11c+ myeloid cells locally and systemically in the model of fibrosis induced by bleomycin repetitions, or even in the case of non-resolving fibrosis such as silicosis, will be essential to dissect more precisely the role of these cells during the fibrogenesis process, and we are interested in exploring this further. In conclusion, the systemic depletion of CD11c+ cells during bleomycin administration confers protection against inflammation and fibrosis, underscoring the significance of myeloid cells expressing F4/80-MHCII+CD11c+ DCs and F4/80+MHCII+CD11c+ MΦ orchestrating the inflammatory milieu within the lungs, potentially as a source of cytokines sustaining pulmonary chronic inflammation leading to progressive fibrosis and mortality.

Acknowledgments:

RCR is grateful to the Conselho Nacional de Desenvolvimento Científico e Tecnológico (CNPq, Brazil) under Bolsa PQ (Grant Agreement No. 309810/2017-5, No. 312839/2020-0, No. 313839/2023-9), and edital Universal 01/2016 - Faixa A/CNPq Grant Agreement No. 422260/2016-9; the Fundação de Amparo a Pesquisa de Minas Gerais (FAPEMIG) Edital Universal under Grant Agreement No. APQ-02571-21 and Rede Mineira de Pesquisa Translacional em Imunobiológicos e Biofármacos no Câncer – REMITRIBIC under Grant Agreement No. RED-00031-21; the Coordenação de Aperfeiçoamento de Pessoal de Nível Superior (CAPES) Programa CAPES-PrInt/UFMG edital 05/2022, and Le Studium Loire Valley – Institute for Advanced Studies (LE STUDIUM Visiting Researcher/ARD CVL Biopharmaceuticals 2023). This work was supported by Centre National de Recherche Scientific (CNRS) and the European Regional Development Fund (FEDER ExposomeInflam). Illustrations were created with BioRender.com.

Author contribution: Conceptualization RCR and FSM designed research. GAOL, BHFL, CSF, ACP, FMS performed experiments. GAOL, BHFL, CSF ACP, GDC, FMS analyzed data. GAOL, BHFL, CSF and RCR wrote the manuscript. FSM provided the CD11c+DTR mice. BR, MMT and FSM contributed with the manuscript revision. All authors have read and agreed to the published version of the manuscript.

Conflict of interest statement: The authors declare no conflicts of interest.

References:

1. White ES, Lazar MH, Thannickal VJ. Pathogenetic mechanisms in usual interstitial pneumonia/idiopathic pulmonary fibrosis. *J Pathol* 2003; **201**: 343-354.
2. American Thoracic Society. Idiopathic pulmonary fibrosis: diagnosis and treatment. International consensus statement. American Thoracic Society (ATS), and the European Respiratory Society (ERS). *Am J Respir Crit Care Med* 2000; **161**: 646-664.
3. Frankel SK, Schwarz MI. Update in idiopathic pulmonary fibrosis. *Curr Opin Pulm Med* 2009; **15**: 463-469.
4. Maher TM, Wells AU, Laurent GJ. Idiopathic pulmonary fibrosis: multiple causes and multiple mechanisms? *Eur Respir J* 2007; **30**: 835-839.
5. Raghu G, Weycker D, Edelsberg J, Bradford WZ, Oster G. Incidence and prevalence of idiopathic pulmonary fibrosis. *Am J Respir Crit Care Med* 2006; **174**: 810-816.
6. Thannickal VJ, Flaherty KR, Hyzy RC, Lynch JP, 3rd. Emerging drugs for idiopathic pulmonary fibrosis. *Expert Opin Emerg Drugs* 2005; **10**: 707-727.
7. Bouros D, Antoniou KM. Current and future therapeutic approaches in idiopathic pulmonary fibrosis. *Eur Respir J* 2005; **26**: 693-702.
8. Scotton CJ, Chambers RC. Molecular targets in pulmonary fibrosis: the myofibroblast in focus. *Chest* 2007; **132**: 1311-1321.
9. Podolanczuk AJ, Thomson CC, Remy-Jardin M, *et al.* Idiopathic pulmonary fibrosis: state of the art for 2023. *Eur Respir J* 2023; **61**.
10. Liu GY, Budinger GRS, Dematte JE. Advances in the management of idiopathic pulmonary fibrosis and progressive pulmonary fibrosis. *BMJ* 2022; **377**: e066354.
11. Pitre T, Mah J, Helmeczi W, *et al.* Medical treatments for idiopathic pulmonary fibrosis: a systematic review and network meta-analysis. *Thorax* 2022; **77**: 1243-1250.
12. Moss BJ, Ryter SW, Rosas IO. Pathogenic Mechanisms Underlying Idiopathic Pulmonary Fibrosis. *Annu Rev Pathol* 2022; **17**: 515-546.
13. Gauldie J. Pro: Inflammatory mechanisms are a minor component of the pathogenesis of idiopathic pulmonary fibrosis. *Am J Respir Crit Care Med* 2002; **165**: 1205-1206.
14. Strieter RM. Con: Inflammatory mechanisms are not a minor component of the pathogenesis of idiopathic pulmonary fibrosis. *Am J Respir Crit Care Med* 2002; **165**: 1206-1207; discussion 1207-1208.
15. Selman M, Pardo A. The epithelial/fibroblastic pathway in the pathogenesis of idiopathic pulmonary fibrosis. *Am J Respir Cell Mol Biol* 2003; **29**: S93-97.
16. Ward PA, Hunninghake GW. Lung inflammation and fibrosis. *Am J Respir Crit Care Med* 1998; **157**: S123-129.
17. Selman M, Pardo A, Kaminski N. Idiopathic pulmonary fibrosis: aberrant recapitulation of developmental programs? *PLoS Med* 2008; **5**: e62.
18. Willis BC, duBois RM, Borok Z. Epithelial origin of myofibroblasts during fibrosis in the lung. *Proc Am Thorac Soc* 2006; **3**: 377-382.
19. Patel VI, Metcalf JP. Airway Macrophage and Dendritic Cell Subsets in the Resting Human Lung. *Crit Rev Immunol* 2018; **38**: 303-331.
20. Kopf M, Schneider C, Nobs SP. The development and function of lung-resident macrophages and dendritic cells. *Nat Immunol* 2015; **16**: 36-44.
21. Vermaelen K, Pauwels R. Pulmonary dendritic cells. *Am J Respir Crit Care Med* 2005; **172**: 530-551.
22. Kulle A, Thanabalasuriar A, Cohen TS, Szydlowska M. Resident macrophages of the lung and liver: The guardians of our tissues. *Front Immunol* 2022; **13**: 1029085.
23. Liu H, Jakubzick C, Osterburg AR, *et al.* Dendritic Cell Trafficking and Function in Rare Lung Diseases. *Am J Respir Cell Mol Biol* 2017; **57**: 393-402.

24. Aegerter H, Lambrecht BN, Jakubzick CV. Biology of lung macrophages in health and disease. *Immunity* 2022; **55**: 1564-1580.
25. Duan W, Croft M. Control of regulatory T cells and airway tolerance by lung macrophages and dendritic cells. *Ann Am Thorac Soc* 2014; **11 Suppl 5**: S306-313.
26. Schittenhelm L, Hilkens CM, Morrison VL. beta(2) Integrins As Regulators of Dendritic Cell, Monocyte, and Macrophage Function. *Front Immunol* 2017; **8**: 1866.
27. Marchal-Somme J, Uzunhan Y, Marchand-Adam S*, et al.* Dendritic cells accumulate in human fibrotic interstitial lung disease. *Am J Respir Crit Care Med* 2007; **176**: 1007-1014.
28. Marchal-Somme J, Uzunhan Y, Marchand-Adam S*, et al.* Cutting edge: nonproliferating mature immune cells form a novel type of organized lymphoid structure in idiopathic pulmonary fibrosis. *J Immunol* 2006; **176**: 5735-5739.
29. Li X, Mara AB, Musial SC*, et al.* Coordinated chemokine expression defines macrophage subsets across tissues. *Nat Immunol* 2024.
30. Russo RC, Quesniaux VFJ, Ryffel B. Homeostatic chemokines as putative therapeutic targets in idiopathic pulmonary fibrosis. *Trends Immunol* 2023; **44**: 1014-1030.
31. Zhang-Hoover J, Stein-Streilein J. Tolerogenic APC generate CD8+ T regulatory cells that modulate pulmonary interstitial fibrosis. *J Immunol* 2004; **172**: 178-185.
32. Upham JW, Stick SM. Interactions between airway epithelial cells and dendritic cells: implications for the regulation of airway inflammation. *Curr Drug Targets* 2006; **7**: 541-545.
33. Kitamura H, Cambier S, Somanath S*, et al.* Mouse and human lung fibroblasts regulate dendritic cell trafficking, airway inflammation, and fibrosis through integrin alphavbeta8-mediated activation of TGF-beta. *J Clin Invest* 2011; **121**: 2863-2875.
34. Florez-Sampedro L, Song S, Melgert BN. The diversity of myeloid immune cells shaping wound repair and fibrosis in the lung. *Regeneration (Oxf)* 2018; **5**: 3-25.
35. Wynn TA, Vannella KM. Macrophages in Tissue Repair, Regeneration, and Fibrosis. *Immunity* 2016; **44**: 450-462.
36. Vannella KM, Wynn TA. Mechanisms of Organ Injury and Repair by Macrophages. *Annu Rev Physiol* 2017; **79**: 593-617.
37. Tsoumakidou M, Karagiannis KP, Bouloukaki I, Zakynthinos S, Tzanakis N, Siafakas NM. Increased bronchoalveolar lavage fluid CD1c expressing dendritic cells in idiopathic pulmonary fibrosis. *Respiration* 2009; **78**: 446-452.
38. Serezani APM, Pascoalino BD, Bazzano JMR*, et al.* Multiplatform Single-Cell Analysis Identifies Immune Cell Types Enhanced in Pulmonary Fibrosis. *Am J Respir Cell Mol Biol* 2022; **67**: 50-60.
39. Taylor ML, Noble PW, White B, Wise R, Liu MC, Bochner BS. Extensive surface phenotyping of alveolar macrophages in interstitial lung disease. *Clin Immunol* 2000; **94**: 33-41.
40. Novak CM, Sethuraman S, Luikart KL*, et al.* Alveolar macrophages drive lung fibroblast function during idiopathic pulmonary fibrosis. *Am J Physiol Lung Cell Mol Physiol* 2023.
41. Bantsimba-Malanda C, Marchal-Somme J, Goven D*, et al.* A role for dendritic cells in bleomycin-induced pulmonary fibrosis in mice? *Am J Respir Crit Care Med* 2010; **182**: 385-395.
42. Bocchino M, Zanotta S, Capitelli L, Galati D. Dendritic Cells Are the Intriguing Players in the Puzzle of Idiopathic Pulmonary Fibrosis Pathogenesis. *Front Immunol* 2021; **12**: 664109.
43. Bar-On L, Jung S. Defining in vivo dendritic cell functions using CD11c-DTR transgenic mice. *Methods Mol Biol* 2010; **595**: 429-442.
44. Liu CH, Fan YT, Dias A*, et al.* Cutting edge: dendritic cells are essential for in vivo IL-12 production and development of resistance against Toxoplasma gondii infection in mice. *J Immunol* 2006; **177**: 31-35.

45. Russo RC, Savino B, Mirolo M, *et al.* The atypical chemokine receptor ACKR2 drives pulmonary fibrosis by tuning influx of CCR2(+) and CCR5(+) IFNgamma-producing gammadeltaT cells in mice. *Am J Physiol Lung Cell Mol Physiol* 2018; **314**: L1010-L1025.
46. Russo RC, Alessandri AL, Garcia CC, *et al.* Therapeutic effects of evasin-1, a chemokine binding protein, in bleomycin-induced pulmonary fibrosis. *Am J Respir Cell Mol Biol* 2011; **45**: 72-80.
47. Russo RC, Garcia CC, Barcelos LS, *et al.* Phosphoinositide 3-kinase gamma plays a critical role in bleomycin-induced pulmonary inflammation and fibrosis in mice. *J Leukoc Biol* 2011; **89**: 269-282.
48. Russo RC, Guabiraba R, Garcia CC, *et al.* Role of the chemokine receptor CXCR2 in bleomycin-induced pulmonary inflammation and fibrosis. *Am J Respir Cell Mol Biol* 2009; **40**: 410-421.
49. Campa CC, Silva RL, Margaria JP, *et al.* Inhalation of the prodrug PI3K inhibitor CL27c improves lung function in asthma and fibrosis. *Nat Commun* 2018; **9**: 5232.
50. Russo RC, Garcia CC, Teixeira MM. Anti-inflammatory drug development: Broad or specific chemokine receptor antagonists? *Curr Opin Drug Discov Devel* 2010; **13**: 414-427.
51. Mattos MS, Ferrero MR, Kraemer L, *et al.* CXCR1 and CXCR2 Inhibition by Ladarixin Improves Neutrophil-Dependent Airway Inflammation in Mice. *Front Immunol* 2020; **11**: 566953.
52. Shenderov K, Collins SL, Powell JD, Horton MR. Immune dysregulation as a driver of idiopathic pulmonary fibrosis. *J Clin Invest* 2021; **131**.
53. Borthwick LA, Barron L, Hart KM, *et al.* Macrophages are critical to the maintenance of IL-13-dependent lung inflammation and fibrosis. *Mucosal Immunol* 2016; **9**: 38-55.
54. Ding L, Liu T, Wu Z, *et al.* Bone Marrow CD11c+ Cell-Derived Amphiregulin Promotes Pulmonary Fibrosis. *J Immunol* 2016; **197**: 303-312.
55. Schaberg T, Rau M, Stephan H, Lode H. Increased number of alveolar macrophages expressing surface molecules of the CD11/CD18 family in sarcoidosis and idiopathic pulmonary fibrosis is related to the production of superoxide anions by these cells. *Am Rev Respir Dis* 1993; **147**: 1507-1513.
56. Chakraborty K, Chatterjee S, Bhattacharyya A. Modulation of CD11c+ lung dendritic cells in respect to TGF-beta in experimental pulmonary fibrosis. *Cell Biol Int* 2017; **41**: 991-1000.
57. Bennett CL, Clausen BE. DC ablation in mice: promises, pitfalls, and challenges. *Trends Immunol* 2007; **28**: 525-531.
58. Mann L, Kochupurakkal N, Martin C, *et al.* CD11c.DTR mice develop a fatal fulminant myocarditis after local or systemic treatment with diphtheria toxin. *Eur J Immunol* 2016; **46**: 2028-2042.
59. Gibbons MA, MacKinnon AC, Ramachandran P, *et al.* Ly6Chi monocytes direct alternatively activated profibrotic macrophage regulation of lung fibrosis. *Am J Respir Crit Care Med* 2011; **184**: 569-581.
60. Bryant AJ, Shenoy V, Fu C, *et al.* Myeloid-derived Suppressor Cells Are Necessary for Development of Pulmonary Hypertension. *Am J Respir Cell Mol Biol* 2018; **58**: 170-180.
61. Schindler D, Gutierrez MG, Beineke A, *et al.* Dendritic cells are central coordinators of the host immune response to Staphylococcus aureus bloodstream infection. *Am J Pathol* 2012; **181**: 1327-1337.
62. Milam JE, Erb-Downward JR, Chen GH, *et al.* CD11c+ cells are required to prevent progression from local acute lung injury to multiple organ failure and death. *Am J Pathol* 2010; **176**: 218-226.
63. GeurtsvanKessel CH, Willart MA, van Rijt LS, *et al.* Clearance of influenza virus from the lung depends on migratory langerin+CD11b- but not plasmacytoid dendritic cells. *J Exp Med* 2008; **205**: 1621-1634.

64. van Rijt LS, Jung S, Kleinjan A, *et al.* In vivo depletion of lung CD11c+ dendritic cells during allergen challenge abrogates the characteristic features of asthma. *J Exp Med* 2005; **201**: 981-991.
65. Faria AMC, Reis BS, Mucida D. Tissue adaptation: Implications for gut immunity and tolerance. *J Exp Med* 2017; **214**: 1211-1226.
66. Bachelerie F, Ben-Baruch A, Burkhardt AM, *et al.* International Union of Basic and Clinical Pharmacology. [corrected]. LXXXIX. Update on the extended family of chemokine receptors and introducing a new nomenclature for atypical chemokine receptors. *Pharmacol Rev* 2014; **66**: 1-79.
67. Pokhreal D, Crestani B, Helou DG. Macrophage Implication in IPF: Updates on Immune, Epigenetic, and Metabolic Pathways. *Cells* 2023; **12**.
68. Arai T, Abe K, Matsuoka H, *et al.* Introduction of the interleukin-10 gene into mice inhibited bleomycin-induced lung injury in vivo. *Am J Physiol Lung Cell Mol Physiol* 2000; **278**: L914-922.
69. Gasse P, Mary C, Guenon I, *et al.* IL-1R1/MyD88 signaling and the inflammasome are essential in pulmonary inflammation and fibrosis in mice. *J Clin Invest* 2007; **117**: 3786-3799.
70. Wilson MS, Madala SK, Ramalingam TR, *et al.* Bleomycin and IL-1beta-mediated pulmonary fibrosis is IL-17A dependent. *J Exp Med* 2010; **207**: 535-552.
71. Papiris SA, Tomos IP, Karakatsani A, *et al.* High levels of IL-6 and IL-8 characterize early-on idiopathic pulmonary fibrosis acute exacerbations. *Cytokine* 2018; **102**: 168-172.
72. Bergeron A, Soler P, Kambouchner M, *et al.* Cytokine profiles in idiopathic pulmonary fibrosis suggest an important role for TGF-beta and IL-10. *Eur Respir J* 2003; **22**: 69-76.
73. Duffield JS, Lupher M, Thannickal VJ, Wynn TA. Host responses in tissue repair and fibrosis. *Annu Rev Pathol* 2013; **8**: 241-276.
74. Warheit-Niemi HI, Huizinga GP, Edwards SJ, *et al.* Fibrotic Lung Disease Alters Neutrophil Trafficking and Promotes Neutrophil Elastase and Extracellular Trap Release. *Immunohorizons* 2022; **6**: 817-834.
75. Ding L, Yang J, Zhang C, Zhang X, Gao P. Neutrophils Modulate Fibrogenesis in Chronic Pulmonary Diseases. *Front Med (Lausanne)* 2021; **8**: 616200.
76. Parsey MV, Tuder RM, Abraham E. Neutrophils are major contributors to intraparenchymal lung IL-1 beta expression after hemorrhage and endotoxemia. *J Immunol* 1998; **160**: 1007-1013.
77. Trachalaki A, Tsitoura E, Mastrodimou S, *et al.* Enhanced IL-1beta Release Following NLRP3 and AIM2 Inflammasome Stimulation Is Linked to mtROS in Airway Macrophages in Pulmonary Fibrosis. *Front Immunol* 2021; **12**: 661811.
78. Tang L, Zhang H, Wang C, Li H, Zhang Q, Bai J. M2A and M2C Macrophage Subsets Ameliorate Inflammation and Fibroproliferation in Acute Lung Injury Through Interleukin 10 Pathway. *Shock* 2017; **48**: 119-129.
79. Tsukui T, Sun KH, Wetter JB, *et al.* Collagen-producing lung cell atlas identifies multiple subsets with distinct localization and relevance to fibrosis. *Nat Commun* 2020; **11**: 1920.
80. Adler M, Mayo A, Zhou X, *et al.* Principles of Cell Circuits for Tissue Repair and Fibrosis. *iScience* 2020; **23**: 100841.
81. Degryse AL, Tanjore H, Xu XC, *et al.* Repetitive intratracheal bleomycin models several features of idiopathic pulmonary fibrosis. *Am J Physiol Lung Cell Mol Physiol* 2010; **299**: L442-452.
82. Tan Q, Link PA, Meridew JA, *et al.* Spontaneous Lung Fibrosis Resolution Reveals Novel Antifibrotic Regulators. *Am J Respir Cell Mol Biol* 2021; **64**: 453-464.

Figure legends:

Figure 1. Bleomycin-induced pulmonary inflammation and mice mortality with progressive airway influx of CD11c+ myeloid DCs and macrophages (MΦ) that precedes lung fibrosis. (A) Total leukocyte counts in suspensions from bronchoalveolar lavage fluid (BALF) followed by flow cytometric analysis of myeloid dendritic cells (DCs; F4/80- MHCII+ CD11c+) and macrophages (MΦ; F4/80+ MHCII+ CD11c+) in bleomycin-treated mice compared to PBS-instilled control wild-type (WT) mice. Data are presented for days 3, 7, and 14 post-instillation, illustrating increased numbers of total airway leukocytes starting from day 3, peaking at day 7, and remaining elevated until day 14. Myeloid DCs increase from day 3 to 14, while MΦ increases from day 7 to 14. (B) Survival curve (n= 20 for bleomycin group and n=5 PBS-instilled control group) indicating pronounced mortality in bleomycin-treated mice after 7 days, coinciding with increased influx of myeloid DCs and MΦ into the airways. (C) Quantification of hydroxyproline content in lung tissue 21 days post-bleomycin instillation, demonstrating significantly elevated levels indicative of extensive pulmonary fibrosis compared to PBS-instilled control mice. (D) Gomori's Trichrome staining of lung sections 21 days post-bleomycin instillation, depicting the dense and diffuse pattern of fibrosis (green), with notable airway collapse in bleomycin-treated mice compared to controls. Scale bars represent 100 µm. (E) Ashcroft's score of pulmonary fibrosis was 21 days post-bleomycin instillation, illustrating a higher grade of fibrosis in bleomycin-treated mice than in PBS-instilled controls. Data represent mean ± SEM from three independent experiments (n=8 mice per group per time point of FACS). Statistical significance was determined using a one-way ANOVA followed by Tukey post-hoc test, and The Mantel-Cox log-rank test was used to determine differences between survival curves (*p<0.05, **p<0.01, ***p<0.001).

Figure 2. Conditional systemic depletion of CD11c+ myeloid cells attenuates airway leukocyte influx, with low DCs and Mϕ numbers 7 days after bleomycin instillation in mice (day 7). CD11c-DTR-Tg mice were systemically depleted of CD11c+ myeloid cells prior to intranasal bleomycin instillation (A). Results demonstrate increased airway leukocyte influx in wild-type (WT) mice after 7 days. Leukocyte infiltration, Total cells (B), macrophages, lymphocytes, and neutrophils (C) into the airways after 7 days in bronchoalveolar lavage (BAL) by cytospin preparations. (D) Flow cytometry analysis of CD4+ lymphocytes (CD3+CD4+), CD8+ lymphocytes (CD3+CD8+), γδ lymphocytes (CD3+TCRγδ+). (E) Flow cytometry analysis of neutrophils (GR1+F4/80-), DCs (F4/80- MHCII+ CD11c+), and macrophages (F4/80+ MHCII+ CD11c+). Data represent mean ± SEM from three independent experiments (n=8 for bleomycin group for WT and CD11c-DTR-Tg mice, and n=5 PBS-instilled control WT and CD11c-DTR-Tg mice group for this time point). Statistical significance was determined using a one-way ANOVA followed by Tukey post-hoc test (*p<0.05, **p<0.01, ***p<0.001).

Figure 3. Systemic CD11c+ Macrophages and DCs depletion protects mice from lung inflammation induced by Bleomycin (day 7). Histopathological analysis of lung tissue following 7 days of bleomycin instillation. (A) Inflammation score based on H&E-stained lung slices depicting lung vascular inflammation, parenchymal leukocyte infiltration, and airway inflammation in wild-type (WT) mice, compared to conditional systemic depletion of CD11c+ cells using CD11c-DTR-Tg mice. (B) The multifocal pattern of lung inflammation observed in WT mice contrasted with increased preserved lung areas in CD11c-DTR-Tg mice. Scale bars represent 100 µm. Overall scores depict the severity of tissue injury and inflammation. Data represent mean ± SEM from three independent experiments (n=8 for bleomycin group for WT and CD11c-DTR-Tg mice, and n=5 PBS-instilled control WT and CD11c-DTR-Tg mice group for this time point). Statistical significance was determined using Kruskal-Wallis followed by Dunn's test (*p<0.05, **p<0.01, ***p<0.001).

Figure 4. Impact of Systemic CD11c+ Macrophages and DCs on chemokine and cytokine levels and fibrogenic gene expression induced by Bleomycin (day 7). Conditional systemic depletion of CD11c+ myeloid cells using CD11c-DTR-Tg mice resulted in decreased levels of chemokines and cytokines compared to wild-type (WT) mice. (A) Analysis of chemokine levels in bronchoalveolar lavage (BAL) fluid quantified by Enzyme-Linked Immunosorbent Assay (ELISA) following 7 days post-bleomycin instillation. (B) Assessment of inflammatory and fibrogenic cytokine levels in lung homogenates. Proinflammatory cytokines IL-1β and IL-6, pro-resolutive cytokine IL-10, and pro-fibrogenic cytokine TGF-β1 were quantified by Enzyme-Linked Immunosorbent Assay (ELISA). (C) Evaluation of mRNA expression related to fibrogenesis via real-time PCR from lung tissue. Expression of Collagen-1α1 (Col1a1) and α-Smooth Muscle Actin (Acta2) mRNA transcripts, indicative of fibroblast activity and extracellular matrix (ECM) production. Data represent mean ± SEM from three independent experiments (n=8 for bleomycin group for WT and CD11c-DTR-Tg mice, and n=5 PBS-instilled control WT and CD11c-DTR-Tg mice group for this time point).

Statistical significance was determined using a one-way ANOVA followed by Tukey post-hoc test (*p<0.05, **p<0.01, ***p<0.001).

Figure 5. Systemic CD11c+ Macrophages and DCs depletion protects mice from pulmonary fibrosis and mortality induced by Bleomycin (day 21). Conditional systemic depletion of CD11c+ myeloid cells using CD11c-DTR-Tg mice was compared to wild-type (WT) mice. (A) Collagen accumulation in lung tissue, measured by hydroxyproline content, 21 days post-bleomycin instillation. (B) Assessment of pulmonary fibrosis using Ashcroft fibrosis scores on Gomori's Trichrome stained lung sections. (C) Gomori's Trichrome staining of lung sections 21 days post-bleomycin instillation, illustrating the dense and diffuse pattern of fibrosis (green), with notable airway collapse in bleomycin-treated WT mice compared to systemic CD11c+ depleted mice (CD11c-DTR-Tg+DTx i.p.). Scale bars represent 100 μm. (D) Survival analysis of mice 21 days post-bleomycin instillation (n=20 for WT and CD11c-DTR-Tg bleomycin groups). Wild-type (WT) mice showed 90% mortality, whereas CD11c-DTR-Tg mice with early depletion of CD11c+ myeloid cells achieved a 60% survival rate. Data represent mean ± SEM from three independent experiments (n=8 for bleomycin group for WT and CD11c-DTR-Tg mice, and n=5 PBS-instilled control WT and CD11c-DTR-Tg mice group for this time point). Statistical significance was determined using a one-way ANOVA followed by a Tukey post-hoc test. The Mantel-Cox log-rank test was used to determine differences between survival curves (*p<0.05, **p<0.01, ***p<0.001).

Figure 6. Local CD11c+ Macrophages and DCs airway depletion enhances mice mortality with exacerbated neutrophilic airway inflammation related to increased CD11c+ Macrophages and DCs influx induced by Bleomycin (day 7). Conditional local depletion of CD11c+ myeloid cells using CD11c-DTR-Tg mice was compared to wild-type (WT) mice (A). Mortality rate following bleomycin instillation in CD11c-DTR-Tg mice with conditional local depletion of CD11c+ myeloid cells (B) using diphtheria toxin (DTx) intranasal (i.n.) (n=20 for WT and CD11c-DTR-Tg mice bleomycin groups). Bleomycin induced 100% mortality in CD11c-DTR-Tg mice, compared to a 60% survival rate in wild-type (WT) mice 8 days post-instillation. (C) Total leukocyte, macrophage, lymphocyte, and neutrophil numbers in cytospin preparations with bronchoalveolar lavage (BAL) fluid 7 days post-bleomycin instillation. (D) Flow cytometry analysis of CD4+ lymphocytes (CD3+CD4+), CD8+ lymphocytes (CD3+CD8+), γδ lymphocytes (CD3+TCRγδ+), and (E) neutrophils (GR1+F4/80-), DCs (F4/80- MHCII+ CD11c+), and macrophages (F4/80+ MHCII+ CD11c+) in WT mice and CD11c-DTR-Tg mice with local CD11c+ depletion. Data represent mean ± SEM from three independent experiments (n=8 for bleomycin group for WT and CD11c-DTR-Tg mice, and n=5 PBS-instilled control WT and CD11c-DTR-Tg mice group for this time point). Statistical significance was determined using a one-way ANOVA followed by a Tukey post-hoc test. The Mantel-Cox log-rank test was used to determine differences between survival curves (*p<0.05, **p<0.01, ***p<0.001).

Figure 7. Impact of local CD11c+ Macrophages and DCs airway depletion on chemokine and cytokine levels, and fibrogenic gene expression in bleomycin-induced lung inflammation in mice (day 7). Conditional local depletion of CD11c+ myeloid cells using CD11c-DTR-Tg mice was compared to wild-type (WT) mice. Bleomycin-induced increased CCL4 and IL-1β pulmonary levels in CD11c-DTR-Tg compared to WT. (A) Analysis of chemokine levels in bronchoalveolar lavage (BAL) fluid quantified by Enzyme-Linked Immunosorbent Assay (ELISA) following 7 days post-bleomycin instillation. (B) Assessment of inflammatory and fibrogenic cytokine levels in lung homogenates. Proinflammatory cytokines IL-1β and IL-6, pro-resolutive cytokine IL-10, and pro-fibrogenic cytokine TGF-β1 were quantified by Enzyme-Linked Immunosorbent Assay (ELISA). (C) Evaluation of mRNA expression related to fibrogenesis via real-time PCR from lung tissue. Expression of Collagen-1α1 (Col1a1) and α-Smooth Muscle Actin (Acta2) mRNA transcripts, indicative of fibroblast activity and extracellular matrix (ECM) production. Data represent mean ± SEM from three independent experiments (n=8 for bleomycin group for WT and CD11c-DTR-Tg mice, and n=5 PBS-instilled control WT and CD11c-DTR-Tg mice group for this time point). Statistical significance was determined using a one-way ANOVA followed by Tukey post-hoc test (*p<0.05, **p<0.01, ***p<0.001).

Figure 8. Local CD11c+ Macrophages and DCs airway depletion leads to increased neutrophilic airway inflammation related to CD11c+ Macrophages and DCs influx induced by Bleomycin in mice (day 7). Conditional local depletion of CD11c+ myeloid cells using CD11c-DTR-Tg mice was compared to wild-type (WT) mice. Histopathological analysis of lung tissue following 7 days of bleomycin instillation. (A) Inflammation score based on H&E-stained lung slices depicting lung vascular inflammation, parenchymal leukocyte infiltration, and airway inflammation in wild-type (WT) mice, compared to conditional local depletion of CD11c+ cells using CD11c-DTR-Tg mice. Overall scores depict the severity of tissue injury and inflammation. (B) Assessment of pulmonary fibrosis using

Ashcroft fibrosis scores on Gomori's Trichrome stained lung sections. (C) Representative photomicrography depicting the multifocal pattern of lung inflammation observed by H&E-staining lung slices in WT mice contrasted with increased cellularity and dense areas in CD11c local depleted mice. Scale bars represent 100 μm. (D) Gomori's Trichrome staining of lung sections 7 days post-bleomycin instillation, illustrating the dense and diffuse pattern of fibrosis (green), with notable airway collapse in local CD11c+ depleted mice (CD11c-DTR-Tg+DTx i.n.) challenged with Bleomycin compared to WT mice. Scale bars represent 200 μm. Data represent mean ± SEM from three independent experiments (n=8 for bleomycin group for WT and CD11c-DTR-Tg mice, and n=5 PBS-instilled control WT and CD11c-DTR-Tg mice group for this time point). Statistical significance was determined using Kruskal-Wallis followed by Dunn's test (*$p<0.05$, **$p<0.01$, ***$p<0.001$).
